# The public information needs of COVID-19 vaccine: A study based on online Q&A communities and portals in China

**DOI:** 10.3389/fpsyg.2022.961181

**Published:** 2022-10-10

**Authors:** Lin Wang, Zuquan Xian, Tianyu Du

**Affiliations:** ^1^Chinese Academy of Science and Education Evaluation, Hangzhou Dianzi University, Hangzhou, China; ^2^School of Managment, Tianjin Normal University, Tianjin, China

**Keywords:** online Q&A communities, COVID-19 vaccine, vaccine information needs, data mining, topic model

## Abstract

**Purpose:**

This study analyzes the topic and distribution features of public information needs for the COVID-19 vaccine from Chinese online Q&A communities and portals. It aims to identify the features and differences in public COVID-19 vaccine information needs at different periods.

**Design/Methodology:**

A total of 14,296 questions about the COVID-19 vaccine from four Chinese mainstream online communities and portals were studied following five procedures: data collection, data processing, K-means clustering, LDA topic model analysis, and needs identification.

**Findings:**

The study identified the topical features of public information needs for the COVID-19 vaccine during the first pandemic outbreak, pre-listing period, and post-listing period. It constructed a framework of public vaccine information needs. The information needs can be classified into 8 main categories and 16 subcategories. The eight main categories are vaccination (53.72%), evaluation and impact of other social events (17.90%), vaccine R&D and listing (9.49%), vaccine side effects and countermeasures (5.63%), vaccination necessity (4.98%), vaccine patent exemption (3.26%), vaccination effectiveness (2.94%), and essential knowledge of vaccine (2.08%), where percentage refers to the distribution of information needs data under various categories.

**Implications:**

Online communities and portals should provide dynamic and tailored information services according to changing public vaccine information needs. The public information needs regarding vaccination is prominent and should be addressed first. In the follow-up booster vaccination efforts, government health departments should prioritize susceptible groups, such as overseas students, airport workers, and healthcare workers.

**Originality/Value:**

We built a conceptual framework using data mining techniques and analyzed the COVID-19 vaccine information needs distribution at different time points and among different social groups, focusing on the theme of public information needs for the COVID-19 vaccine. It makes recommendations for government health departments and online platforms to improve the quality of COVID-19 vaccine information services for the public and provide a reference for the vaccination of COVID-19 booster shots.

## Introduction

The COVID-19 pandemic is still spreading throughout the world. The continuous variation in the virus causing COVID-19 has increased its infectivity and viral load, making it difficult for global pandemic prevention and control. This has increased public demand for the COVID-19 vaccine. As of 6 August 2021, a total of 4.36 billion COVID-19 vaccine doses have been administered worldwide (The Novel Coronavirus Pandemic: The Extent of Vaccination Process Around the World in Your Region, 2021). However, this number is still far away from the global population. During the same period, the number of people who have completed the full course of COVID-19 vaccination in China reached 770 million (National Health Commission, 2021), accounting for only half of its total population. As a result, public concern about the COVID-19 vaccine will remain high in the future.

In the digital era, people have been actively obtaining health information through various online channels (Huang et al., 2020; National Health Commission, 2021). According to the 48th Statistical Report on China's Internet Development, China's Internet user base reached 1.011 billion in December 2020, with an Internet penetration rate of 71.6% (China Internet Network Information Center, 2021). During the pandemic, the information needs for the COVID-19 vaccine shifted more to online platforms due to social decline. Moreover, public information needs are reflected by raising questions on relevant online Q&A communities and portals (Williamson et al., 2012; Price and Robinson, 2021). Among them, questions about the COVID-19 vaccine become one of the main contents of pandemic information needs. However, the existing research does not address this. This study studied 14,296 questionnaire data about the COVID-19 vaccine from four mainstream online Q&A communities and portals in China using the K-means clustering algorithm and LDA topic model analysis. Then, we established the public's COVID-19 vaccine information needs framework under the online community platform and explored the characteristics of COVID-19 vaccine information needs distribution. In addition, we discussed the COVID-19 vaccine information needs of different social groups. Our study aimed to improve the understanding of the patterns and features of the public information needs for the COVID-19 vaccine. It provides a reference for governments and relevant stakeholders to assess the status quo better, optimizes their service quality (Wu and Liu, 2018), and helps them address the problems in decision-making about COVID-19 health.

## Literature review

### Research on public health information needs

The existing studies on the information needs in online Q&A communities and portals focused on information needs in specific domains. For people with diabetes, Jin and Xu (2015) investigated the topic features of their information needs by content analysis of online question data and referencing existing diabetes information needs categories. They concluded that daily disease management, diagnosis, and treatment were the most important aspects of diabetes health information needs. In terms of the HPV vaccine, Huang and Zhou (2020) studied the questioning records about the HPV vaccine in the “Zhihu” community by content analysis. They classified the HPV vaccine information needs into vaccine effectiveness, side effects, vaccination knowledge, and vaccination channels. Moreover, as far as oncology and cancer are concerned, Özbayir et al. (2011) explored the information needs of typical meningioma patients at 1, 3, 6, and 12 months after surgery. The results displayed that diet, physical therapy, rehabilitation, quick thinking difficulties, fasting, headache, irritability, personality change, fatigue, driving, and deep vein thrombosis are the most concerned information of patients. Rutten et al. (2005) collected and analyzed 112 articles on cancer patients' information needs published from 1980 to 2003. The analysis demonstrated that patients' information needs are focused on disease stages, treatment options, and treatment side effects during diagnosis and treatment and concentrate on treatment information and recovery information after treatment. Oh et al. (2016) used cancer-related question data on the “Yahoo! Answers” Q&A platform to conduct text mining and derived six cancer information needs categories: demographic, cognitive, emotional, social, situational, and technical. Lu et al. (2019) utilized Latent Semantic Indexing (LSI) model, and MapReduce distributed clustering method to mine 24,305 question data in the oncology section of the “Qiuyi” website. They constructed a framework on information needs and calculated the proportion of each need type, which were treatment (43.3%), pathology and etiology (34.5%), examination (12.1%), postoperative (7.0%), and prevention (3.1%). In addition, Thelwall (2021) examined international differences in vaccination against COVID-19 by analyzing tweets about the vaccine, finding that “differing extents to which non-government scientific experts are important to national vaccination discussions” played a role and highlighted the need for international sharing of supplies.

These studies primarily looked into the information needs of the general public and specific communities in specific fields. During the COVID-19 pandemic, the public's need for COVID-19 vaccine information was high, but no research on the topic has been found. In addition, studies on health information needs mainly employ traditional research methods, such as questionnaire surveys, interviews, and content analysis, which have several drawbacks. For example, the number of research subjects is limited, and the research results may be affected by unrepresentative samples. In this study, we applied the web crawler as the data collection method to ensure enough sample data for extensive data analysis. It has been proved as an effective way of obtaining more universal research results in social sciences (Zhao and Li, 2022). The findings of this study can illustrate the characteristics of the COVID-19 vaccine information needs of the general public in China and help the government and stakeholders improve information service quality and health policies.

### Research on influencing factors of vaccination intention

Influencing factors on vaccination intention is a common topic in vaccine information needs. In the field of the HPV vaccine, Galvin et al. (2021) evaluated the correlation between HPV vaccine information quality and users' vaccination by testing the exposure of users and their children to HPV vaccine information on social media. They found that increasing the number of positive messages and information credibility promotes users' willingness to receive the vaccine. Zhou (2020) set up two scenario experiments and administered questionnaires to 200 randomly selected citizens. The results demonstrated that the presentation of negative information about vaccines had no significant correlation with the willingness to vaccinate against HPV. However, the psychological risk of infection and disease severity were significantly related to vaccination intention. Kwon et al. (2010) found that the influencing factors of public intention to vaccinate against influenza A (H1N1) mainly included the fear of influenza A (H1N1), the likelihood of infecting the virus, prioritization in the production of novel influenza vaccines, and the effectiveness of the vaccine. In addition, some scholars have studied the influencing factors of vaccination intention for different public groups. For example, Nikula et al. (2009) conducted focus group discussions and interviews with 40 healthcare professionals, students, and clients and reported that vaccinators' professional conduct, education, client conduct, and vaccination environment impact vaccination. Kalaij et al. (2021) analyzed 16 relevant research articles in three databases (PubMed, Scopus, and Cochrane) on factors associated with childhood vaccination in Southeast Asia. They identified that parental, personal-related, children and family status-related, socioeconomic, and healthcare-related factors strongly influence subjects' immunization. Huang et al. (2018) adopted a multi-stage random sampling method to select 652 parents whose children were aged 3–10 years old in Nanhai District, Foshan City, China. They used a face-to-face questionnaire to analyze parents' cognition of the varicella vaccination and its influencing factors. They concluded that children's age, parent's education level, children's history of varicella, acceptable vaccine price, and vaccine information accessibility were the factors influencing the vaccination rate of children.

The above-mentioned studies have discussed the public's willingness to vaccinate against various diseases and their influencing factors. However, until now, previous studies have not addressed the public's willingness to vaccinate against COVID-19. It is believed that meeting information needs can alleviate information and promote vaccination willingness in public health emergencies (Maire et al., 2021). Therefore, we will analyze the levels and features of the public's COVID-19 vaccine information needs at different stages and divide such needs into several categories by constructing the public's COVID-19 vaccine information need framework. Expected findings will contribute to research on factors influencing COVID-19 vaccination willingness.

## Research questions

The public information needs of the COVID-19 vaccine are examined based on Chinese mainstream online Q&A communities and portals. The study mainly focuses on the following questions: (1) What is the classification framework of public COVID-19 vaccine information needs under Chinese online Q&A communities and portals? What is the proportion of each need category? (2) What are the distribution characteristics of public COVID-19 vaccine information needs (including topic distribution and temporal distribution)? What social phenomena do these characteristics reflect? (3) What about the distribution of COVID-19 vaccine information needs among different social groups? What insights could we gain from it? Through the above exploration, we can effectively grasp the patterns and characteristics of public information needs for the COVID-19 vaccine and provide reference and guidance for governments and relevant operators to understand the status quo better, optimize their service level, and impel the subsequent vaccination of COVID-19 booster shots.

## Methods

**Figure 1:**
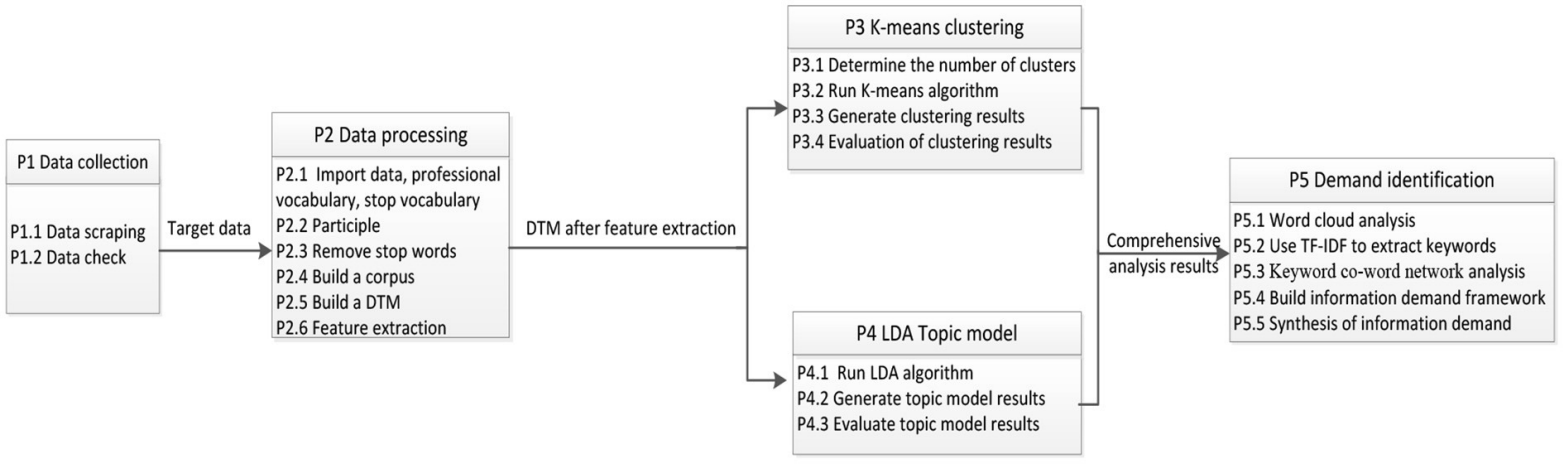
Procedure for analyzing the public COVID-19 vaccine information needs.

In this study, we analyze the data on the public information needs of the COVID-19 vaccine from four online Q&A communities and portals by utilizing the text mining method. The specific analysis procedure is displayed in Figure 1. It includes five steps: data collection and examination, data processing, converting the raw data into a Document Term Matrix (DTM) that can be recognized and processed by computers, analyzing the DTM by K-means clustering and LDA topic model, and synthesizing the results generated by both and identifying the public information needs of COVID-19 vaccine (Mi et al., 2021).

### Data collection and processing

#### Data source and collection

We collected data on the COVID-19 vaccine information needs from the platforms of “Zhihu,” “Baidu Post,” “39Health.com”, and “Chunyu Doctor.” The “Zhihu” and “Baidu Post” platforms are the mainstream online Q&A communities, and “39Health.com” and “Chunyu Doctor” are two mainstream healthcare portals in China. The four platforms together have over 1.03 billion registered users, of which over 503 million are active. The data on the platform can fairly reflect the information requirements of the Chinese public. Therefore, the questions on these platforms reflect the public information needs regarding the COVID-19 vaccine. We constructed the dataset as 14,296 questions along with their number of answers, the number of followers, and questioning time on these four platforms from 23 January 2020 to 15 July 2021. We searched these data using Octopus collector version V8.1 (https://www.bazhuayu.com) with the keyword “COVID vaccine” to crawl and de-duplicate the data. The data were then saved in a CSV file.

#### Data processing

We imported the data, professional thesaurus, and stop words list in the data processing step. We programmed using the R language, used the RSTUDIO compiler environment, and imported the data through the “read.csv().” Baidu medical thesaurus (https://shurufa.baidu.com/dict_list?cid=217), which contains 65,462 words, was adopted. We also adopted the HIT (Harbin Institute of Technology) stop words list (https://github.com/goto456/stopwords/blob/master/hit_stopwords.txt), which contains 676 stop words. The Baidu medical thesaurus and HIT stop word list are widely used by Chinese scholars (Wu, 2013; Xu, 2013; Yang, 2013; Hu, 2018; Li, 2019; Zhu, 2020). Then, we carried out word segmentation. Chinese word segmentation methods mainly include lexicon-based and statistics-based word segmentation (Zong et al., 2019). We adopted the lexicon-based word segmentation method, realizing the word segmentation with the Jiebar package developed in the R language. Stop words had to be removed during this process to ensure that the textual features were extracted correctly. Afterword segmentation, the data types were transformed into the corpus, and the corpus into Document Term Matrix (DTM) by the “DocumentTermMatrix()” method. DTM is a two-dimensional matrix in which the first row represents all feature words in the corpus, the first column represents the serial number of users' question data, and the matrix value represents the co-occurrence frequency of feature words in each document. The initial DTM has many dimensions. To improve the algorithm's running speed and clustering accuracy, feature screening and extraction of the initial DTM are required. The standard methods are principal component analysis (PCA), singular value decomposition (SVD), and manual feature screening. In this study, we set thresholds for word frequency and word length in the DTM to filter features and θ_m_ retained 837 feature words with word frequency higher than 10 and word length longer than one.

### K-means clustering

The clustering algorithm is an unsupervised learning algorithm that studies how to classify objects, including the K-means clustering algorithm, density-based clustering algorithm, hierarchical clustering algorithm, expectation-maximizing clustering algorithm, etc. (Zong et al., 2019). K-means clustering algorithm is a widely used segmentation-based clustering algorithm, which divides the raw data into different clusters by calculating the similarity between data so that the data between clusters are different. In contrast, the data within the clusters are similar. Therefore, the textual data of public COVID-19 vaccine information needs crawled from the four platforms can be effectively clustered by the K-means algorithm, which provides the basis for the subsequent construction of the public COVID-19 vaccine information needs framework.

The K-means clustering algorithm has two key points: the cluster number's determination and the algorithm results' evaluation. The elbow method is a standard method for cluster number determination. According to Equation (1), the cost function curves are plotted for different k values. As the k value increases, the k value corresponding to the elbow of the function curve is the optimal number of clusters. The *u*_*k*_ in the equation is the center coordinate of the kth category (Mi et al., 2021).

(1) $$\begin{array}{l} J=\sum_{1}^{\text{k}}\sum_{i}\in C_{k}|x_{i}-u_{k}{|}^{2} \end{array}$$

The evaluation of algorithm results in this study mainly applies the Silhouette Coefficient (SC) method, a commonly used internal criterion for the evaluation of clustering algorithms, as revealed in Equation (2). *a*(*d*) represents the cohesiveness degree of the cluster to which sample d belongs, *b*(*d*) represents the separation degree of sample d from other clusters, and SC denotes the silhouette coefficient.

(2) $$\begin{array}{l} SC\left(d\right)=\frac{b\left(d\right)-a\left(d\right)}{\max\left\{a\left(d\right),b\left(d\right)\right\}} \end{array}$$

### LDA topic model

**Figure 2:**
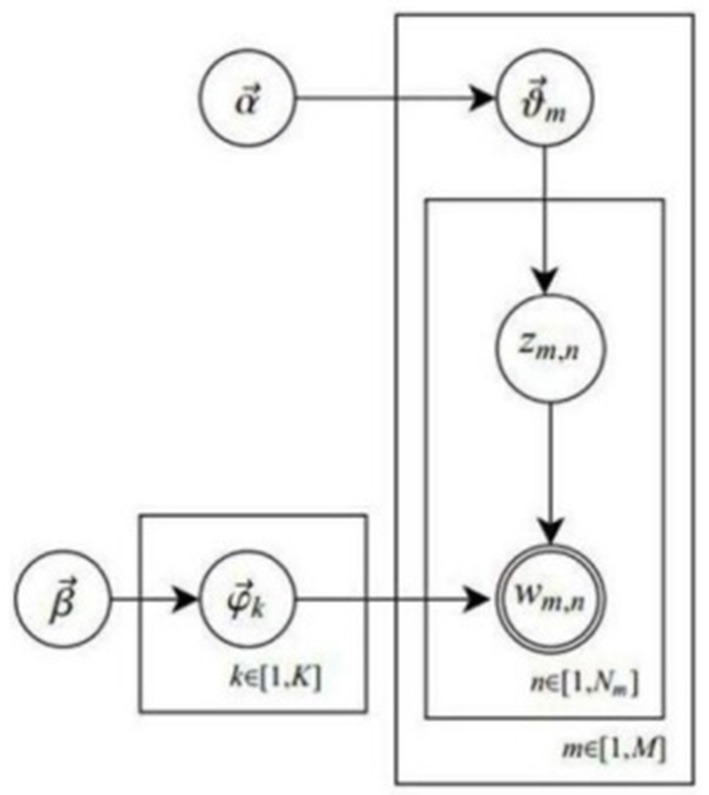
LDA topic model probability diagram.

The latent Dirichlet Allocation (LDA) topic model is proposed based on the Probabilistic Latent Semantic Analysis (PLSA) model, which is also a clustering algorithm. Unlike K-means clustering, LDA is a probability-based algorithm, a three-layer Bayesian model including document, topic, and word (Blei et al., 2003). LDA is one of the most popular models in text mining. On the one hand, it can be used as a dimension reduction tool. After the LDA model is trained, the document can be represented in the topic space. Document processing in word space can thus be done in topic space using the LDA model. On the other hand, collaborative filtering, document similarity calculation, and text segmentation can be accomplished with the parameter estimates of the topic model. In this study, we applied the LDA topic model to analyze the textual data of the public information needs of the COVID-19 vaccine for the subsequent topic discovery. LDA assumes that a “topic-word” distribution parameter is generated for each topic: φ_*k*_ ~ *Dir*(β). A “document-topic” distribution parameter is formed for each document: θ_*m*_ ~ *Dir*(α). The topics for the document in the current location begin to take shape: *z*_*m,n*_ ~ *Cat*(θ_*m*_). Hence, corresponding words for the topic in the current location come into being: ω_*m,n*_ ~ *Cat*(φ_*z*_*m,n*__). The probability diagram of the LDA topic model is illustrated in Figure 2, and the explanation of the parameters is displayed in Table 1 (Zong et al., 2019).

**Table 1:** Explanation of the parameters in LDA topic model probability diagram.

**Symbols**	**Meaning**	**Symbols**	**Meaning**
M	Number of documents	K	Number of topics
V	Number of words (Word list dimension)	α	The prior distribution hyperparameters of θ_*m*_
β	The prior distribution hyperparameters of φ_k_	θ_m_	Topic parameter distribution of the *m-*th document
φ_k_	The words distribution parameters for the k-th topic	*N*_m_	Length of the *m-*th document
z_*m, n*_	The topic corresponding to the *n-*th word of the *m-*th document	*w*_*m, n*_	The lexical term corresponding to the nth word of the *m-*th document
${\text{z}}_{m}={\left\{{\text{z}}_{m,n}\right\}}_{n=1}^{N_{m}}$	The topic sequence corresponding to the *m-*th document	${\text{w}}_{m}={\left\{{\text{w}}_{m,n}\right\}}_{n=1}^{N_{m}}$	The lexical word sequence corresponding to the *m-*th document
$\text{w}={\left\{{\text{w}}_{\text{m}}\right\}}_{m=1}^{M}$	The word sequence corresponding to the document set	$\text{z}={\left\{{\text{z}}_{\text{m}}\right\}}_{m=1}^{M}$	The topic sequence corresponding to the document set

### Needs identification

The results obtained from the K-means clustering and LDA topic model analysis need to be reconsidered from the perspective of information need theory. We comprehensively analyzed the results and referenced the existing classification framework to derive the public COVID-19 vaccine information needs categories. The TF-IDF method extracted the topic keywords from each category's feature words. The “TF” refers to the term frequency, and its calculation is displayed in Equation (3), where *f* (*t, d*) indicates the frequency that the term t appears in document d and ∑_*k*_*f*(*w*_*k*_, *d*) indicates the frequency of all terms that appear in document d. IDF refers to the inverse document frequency. Its calculation is illustrated in Equation (4), where |D| indicates the total number of documents in the document set, and |d ϵ D: t ϵ d| indicates the number of documents containing the word t in the document set (Zhang et al., 2019). TF-IDF means the product of TF and IDF, as detailed in Equation (5). The extracted keywords were analyzed, and a word cloud was drawn. In addition, it was essential to conduct a statistical analysis of the public COVID-19 vaccine information needs pattern. Therefore, the keyword co-word network and bar chart were drawn to visualize the results.

(3) $$\begin{array}{l} \mathrm{tf}\left(t,d\right)=\frac{f\left(t,d\right)}{\sum_{k}f\left(w_{k},d\right)} \end{array}$$

(4) $$\begin{array}{l} \mathrm{idf}\left(t,d\right)=\mathrm{lg}\frac{\left|D\right|}{\left|d\in D:t\in d\right|} \end{array}$$

(5) $$\begin{array}{l} \mathrm{tf}\text{\_}idf\left(t,d\right)=tf\left(t,d\right)\times idf\left(t,d\right) \end{array}$$

## Results

### Basic features of public information needs of COVID-19 vaccine

By crawling and examining the data on the information needs of COVID-19 vaccine from the platforms of “Zhihu,” “Baidu Post,” “39Health.com,” and “Chunyu Doctor” with the keyword of “COVID-19 vaccine”, a total of 14,296 questions, as well as their number of answers, number of followers, and questioning time, were collected. The basic statistics of these data are demonstrated in Table 2.

**Table 2:** Basic statistics of data on the public information needs of COVID-19 vaccine.

**Basic feature**	**Mean value**	**Median value**	**Mode**	**Standard deviation**	**Minimal value**	**Maximum value**
Question length (characters)	22.29	20	15	10.906	5	51
Number of answers (pcs)	14.786	1	0	68.483	0	1,319
Number of followers (pcs)	64.772	3	1	277.592	0	3,714

**Figure 3:**
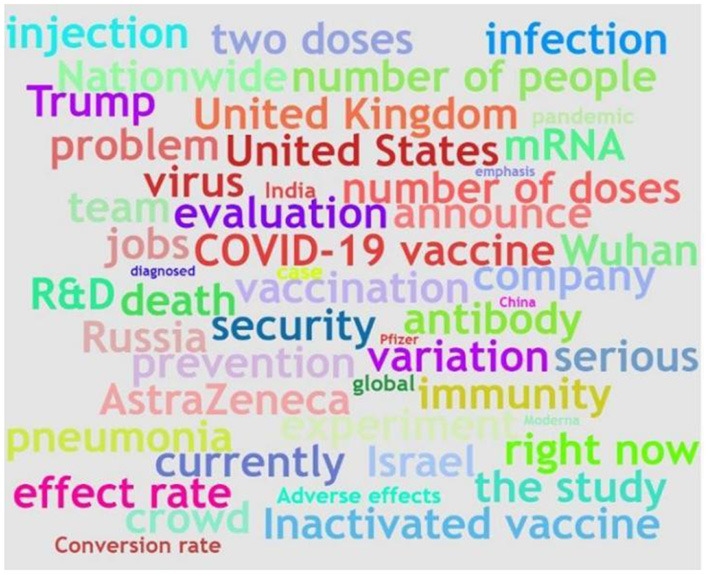
Word cloud diagram.

The statistical data show that the standard deviation of the number of followers is the largest. The standard deviation of the number of answers takes second place, reflecting that the public needs for different types of COVID-19 vaccine information varied greatly. To investigate the focus of the COVID-19 vaccine's public information needs, we set a threshold value of 10 for the number of followers and answers of the question data to extract questions with more than 10 followers and answers. Then, we analyzed these data to obtain a word cloud diagram of the topics with serious concerns and answers, which contained the top 50 topic keywords, as displayed in Figure 3. We can find from the graph that the “COVID-19 vaccine” is the gist of social concerns, and “vaccination,” “research and development,” and “evaluation” are significant aspects of public information needs. In addition, the keyword “experiment” implied that the public follows the vaccine R&D progress by accessing information related to COVID-19 vaccine trials. The keyword “antibody” indicates that the public paid great attention to the concentration of antibodies produced by COVID-19 vaccines, which helps them judge the effectiveness of various vaccines. “AstraZeneca” is a world-renowned COVID-19 vaccine supplier. The public attaching importance to information about “AstraZeneca” meant that the Chinese public was inclined to compare domestic and foreign vaccines to choose the most suitable for them.

We classified the evolution of public vaccine information needs into three stages according to the critical time points of the pandemic development: the first pandemic outbreak period (23.01.2020–08.04.2020), the vaccine pre-listing period (09.04.2020–31.12.2020), and the vaccine post-listing period (01.01.2021–15.07.2021). The division between the pandemic first outbreak period and the vaccine pre-listing period is based on the lifting lockdown time in Wuhan, China. The division between the vaccine pre-listing and post-listing periods relied on when Sinopharm, China's first COVID-19 vaccine R&D and production company, received marketing authorization from the National Medical Products Administration. The public information needs for the COVID-19 vaccine are higher during the first pandemic outbreak period, while it is significantly lower during the vaccine pre-listing period, with an average of only 15.44 questions per day. It indicates that people's lives gradually returned to normal with the overall improvement of China's pandemic prevention and control situation. Hence, the information needs for the COVID-19 vaccine decreased significantly. In contrast, the need for public information increased again in the vaccine post-listing period, reaching a daily average of 32.53 items. This reflects that the availability of the vaccine, the government's vigorous vaccination promotion, and the worsening global pandemic amplified the public information needed for the COVID-19 vaccine.

To better explore the content features of public information needs of COVID-19 vaccine in each period, 15 keywords and their TF-IDF values were extracted from each period in this study. The results are displayed in Table 3. From the table, it can be seen that during the first outbreak period, the public information needs mainly focused on vaccine safety, vaccination necessity, adverse effects of the vaccine, and whether the vaccine could cope with the variation of novel coronavirus. During the pre-listing period, the public primarily needs information about vaccine listing, R&D, and vaccine safety. The types of information that the public mostly demanded during the post-listing period were cautions before and after vaccination, vaccine event evaluation, and adverse effects.

**Table 3:** Keywords of the public information need of COVID-19 vaccine in different periods.

**Outbreak Period**		**Pre-listing Period**		**Post-listing Period**	
**Keywords**	**TF-IDF value**	**Keywords**	**TF-IDF value**	**Keywords**	**TF-IDF value**
COVID-19 Vaccine	0.312	COVID-19 Vaccine	0.306	Vaccinate	0.301
Variation	0.189	Listing	0.256	Vaccination	0.223
Going Abroad	0.177	Success	0.192	After Vaccination	0.098
Suppliers	0.146	R&D	0.184	Viewpoint	0.058
Security	0.115	Necessity	0.068	mRNA	0.052
Necessity	0.056	Viewpoint	0.064	Beijing Institute of Biological Products	0.050
mRNA	0.055	Safety	0.048	Variation	0.050
AstraZeneca	0.041	Large Scale	0.036	Antibodies	0.044
Adverse reactions	0.036	Everyone	0.036	Everyone	0.039
Protection	0.036	mRNA	0.036	Safety	0.038
Others	0.029	Variation	0.036	Necessity	0.038
Vaccinate	0.028	Evaluation	0.034	Going Abroad	0.034
DNA	0.027	Moderna	0.034	Adverse reactions	0.031
Report	0.022	Beijing Institute of Biological Products	0.030	Protection	0.029

**Figure 4:**
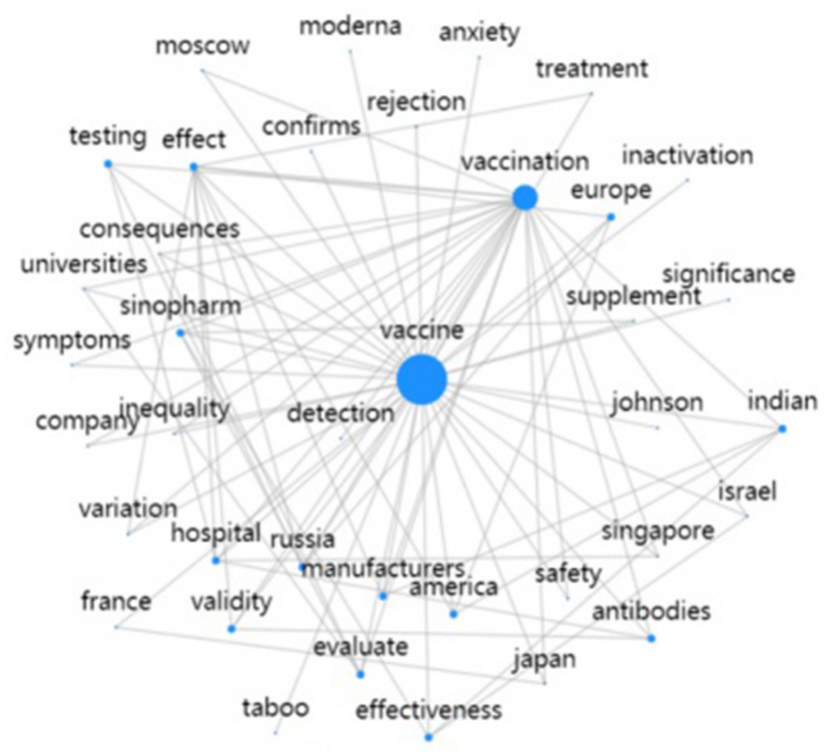
Keyword co-word network.

We translated the Chinese text dataset into English, then put the English data into GooSeeker online software (https://nlp.gooseeker.com/fenci), and drew the keyword co-word network graph by co-word network analysis (Figure 4). The size of blue circles indicates the number of keyword occurrences, and the connecting lines indicate the links between keywords. The graph shows that “vaccine” and “vaccination” are the keywords most closely related to other words. Therefore, the public needed more information about the vaccine effect, the evaluation of vaccine-related events, and the vaccine suppliers. In addition, some keywords represent countries and regions, such as “Japan, America, Europe, Moscow, Singapore, India, and Israel,” indicating that the public is also concerned about social events related to the COVID-19 vaccine in other countries and regions. For example, asking for perspectives on COVID-19 vaccine patent exemption in the United States, asking why Japan did not purchase the Chinese COVID-19 vaccine, and asking for comments on the event that a vaccinated US expert died of COVID-19 infection when he traveled to India.

### Public COVID-19 vaccine information needs framework

**Figure 5:**
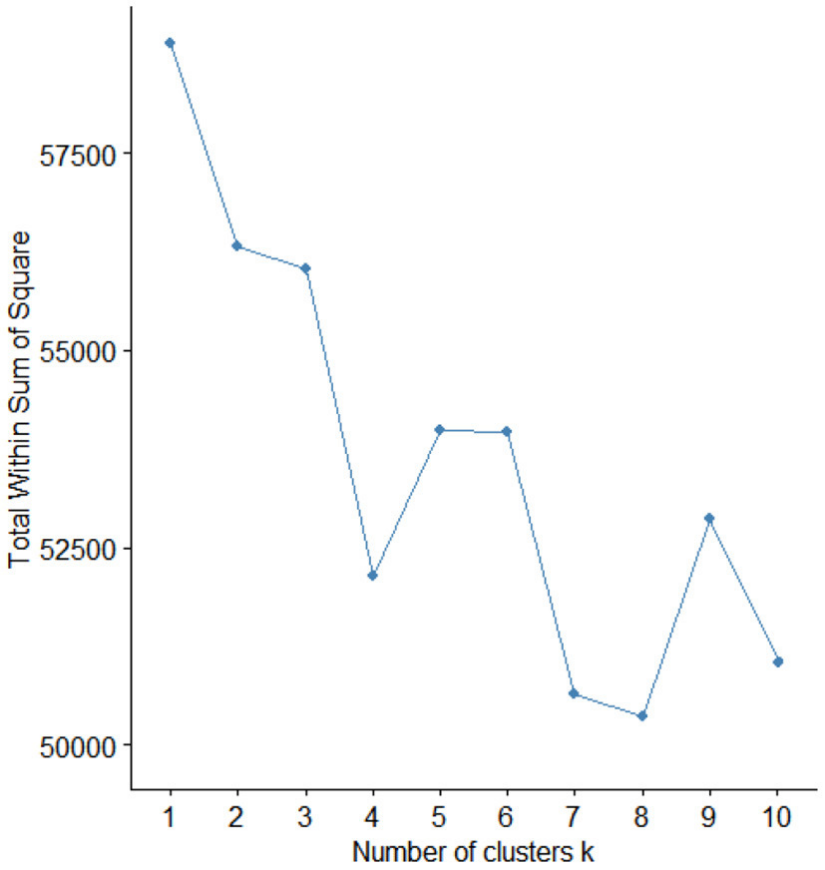
The results of elbow method.

By combining the results of K-mean clustering, the LDA topic model, and the existing health information needs classifications (Tang and Li, 2019), we constructed the public COVID-19 vaccine information needs framework. The main category number in this framework is determined based on the elbow method. The result is illustrated in Figure 5, demonstrating that the number of clusters determined to be the most appropriate is 8. In the same way, the number of subcategories in each main category could be determined. We named and obtained this framework's 8 main categories and 16 subcategories according to the LDA results. We continued to revise the data clustering results through the content analysis method. Since three subcategories of C4 (vaccination necessity), C5 (vaccine effectiveness), and C6 (vaccine side effects and countermeasures) were the focus of public attention, they were adjusted as first-level categories. The proportion of each type of information needed is demonstrated in Table 4.

**Table 4:** The public COVID-19 vaccine information needs a framework.

**Category**	**Subcategories**	**Description**	**Number of questions/time**	**Proportion/%**
C1 Basic knowledge of the vaccine	C1.1 Vaccine mechanism	Asking for the preparation mechanism of COVID-19 vaccine.	82	0.57%
	C1.2 Vaccine effect	Asking how COVID-19 vaccine works.	94	0.66%
	C1.3 Difference in various COVID-19 vaccines	Ask for differences between different types of vaccines, such as adenovirus vector vaccines, inactivated vaccines, and recombinant protein vaccines.	122	0.85%
	Total		298	2.08%
C2 Vaccine R&D and listing	C2.1 Vaccine R&D process	Asking how is COVID-19 vaccine R&D going.	1016	7.11%
	C2.2 Vaccine listing time	Asking when COVID-19 vaccine will go public.	340	2.38%
	Total		1,356	9.49%
C3 Vaccination	C3.1 Vaccination appointment	Asking for information on how, when, and where to make an appointment for COVID-19 vaccination.	522	3.65%
	C3.2 Vaccination fees	Asking for the cost of vaccination against novel coronavirus.	371	2.60%
	C3.3 Vaccine type	Asking what kind of vaccine will be vaccinated.	156	1.09%
	C3.4 Vaccination population confirmation	Asking for the scope of vaccination population and if someone can receive COVID-19 vaccine when he or she has a past medical history, medication history, or physical discomfort symptoms.	2,036	14.24%
	C3.4 Preparation before vaccination	Asking what must be made before vaccination	1,591	11.13%
	C3.5 Cautions after vaccination	Asking what to look for after vaccination.	2,183	15.27%
	C3.6 Vaccination procedure	Asking what the procedure for COVID-19 vaccination is; asking for details of vaccination procedure.	821	5.74%
	Total		7,680	53.72%
C4 Vaccination necessity		Asking for the necessity of vaccination against novel coronavirus.	712	4.98%
C5 Vaccination effectiveness		Asking about the effectiveness of COVID-19 vaccine and comparing the effectiveness of different types of COVID-19 vaccines.	420	2.94%
C6 Vaccination side effects and countermeasures	C7.1 Side effects	Asking for any side effects or adverse reactions to the COVID-19 vaccine.	694	4.85%
	C7.2 Countermeasures	Asking what measures to deal with side effects or adverse reactions after vaccination.	112	0.78%
	Total		806	5.63%
C7 Vaccine patent exemption		Asking about the patent exemption for COVID-19 vaccine.	465	3.26%
C8 Evaluation and impact of other social events	C8.1 Evaluation of other social events	Asking for evaluating other social events regarding the COVID-19 vaccine (e.g., how do you rate the COVID-19 vaccine appointment at the University of Electronic Science and Technology in China?).	2084	14.58%
	C8.2 Impact of other social events	Asking for the impact of other social events regarding COVID-19 vaccine (e.g., what would be the impact if China were to take the lead in developing the COVID-19 vaccine?).	475	3.32%
	Total	2559	17.90%	

As indicated in Table 4, it is apparent that the top three public COVID-19 vaccine information needs are vaccination (53.72%), evaluation and impact of other social events (17.90%), and vaccine R&D and listing (9.49%). The three ones with the smallest share are vaccine effectiveness (2.94%), basic knowledge of the vaccine (2.08%), and vaccine patent exemption (3.26%). Among the subcategories, the top three kinds of information needs are vaccination population confirmation (14.24%), cautions after vaccination (15.27%), and evaluation of other social events (14.58%). On the other hand, the three ones with the smallest share are the vaccine mechanism (0.57%), the vaccine effect (0.66%), and countermeasures (0.78%). These results indicate that the public had a great interest in the cautions before and after vaccination and inquiries about vaccine R&D process and listing time, but less concerned about the basic knowledge of vaccines. In addition, the public information need regarding the countermeasures for vaccine side effects is low, reflecting that there are very few side effects after vaccination and confirming the safety and reliability of the Chinese COVID-19 vaccine.

### COVID-19 vaccine information needs of different social groups

**Figure 6:**
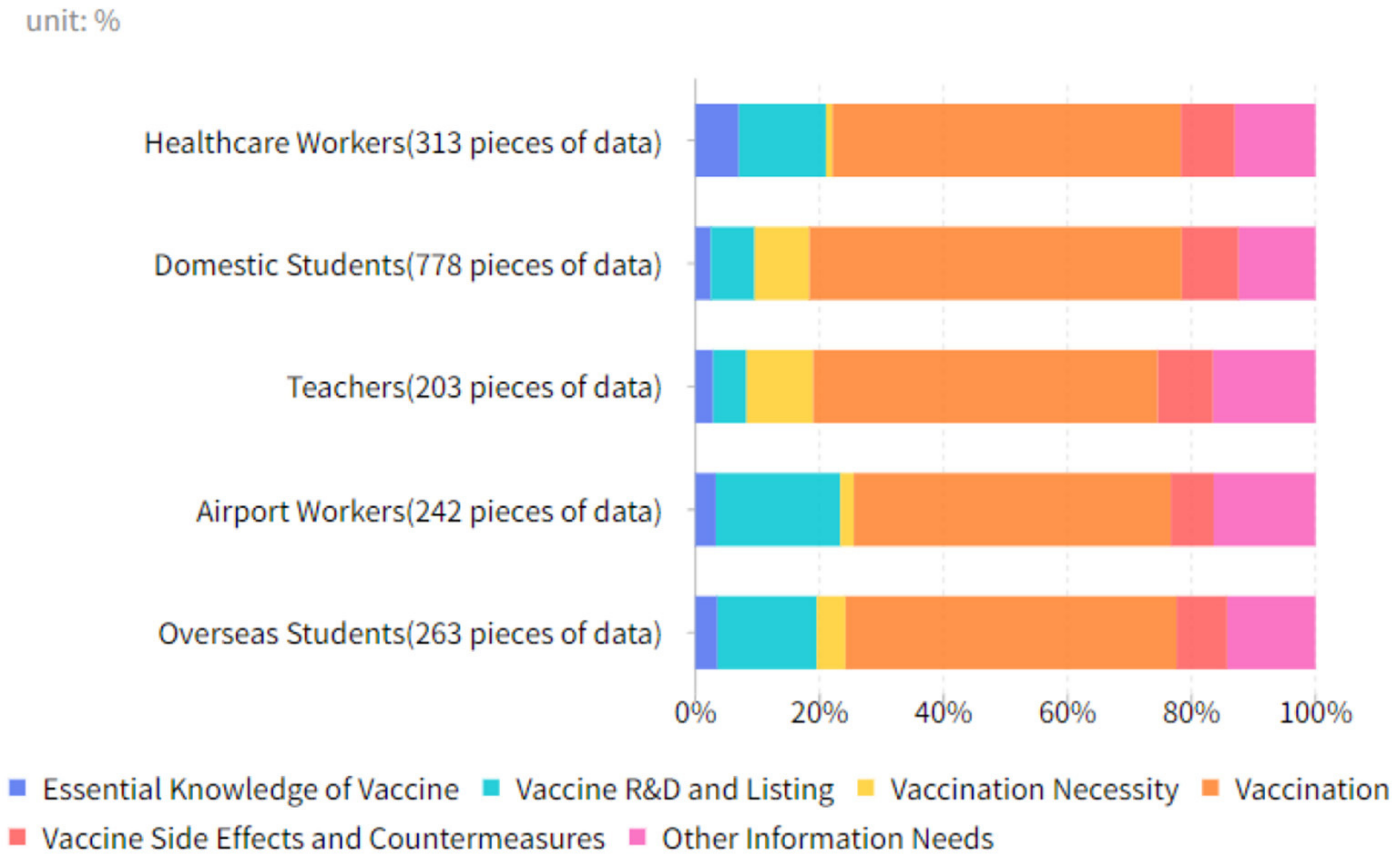
COVID-19 vaccine information needs of different social groups during the pandemic.

This study found 1,799 questions about the COVID-19 vaccine with keywords of overseas students, airport workers, teachers, domestic students, and healthcare workers. We obtained COVID-19 vaccine information needs distribution for different social groups through statistical analysis, as demonstrated in Figure 6. It illustrated that the proportion of information needed for vaccination is high among all groups. However, overseas students, airport workers, and healthcare workers have higher needs for information about vaccine R&D and listing relatively. In comparison, teachers and domestic students have relatively higher needs for information about vaccination necessity.

## Discussion

This study uses data mining to investigate the Chinese public information needs for the COVID-19 vaccine. We innovatively divided COVID-19 vaccine information needs into three periods: the first pandemic outbreak period (23.1.2020–08.04.2020), the vaccine pre-listing period (09.04.2020–31.12.2020), and the vaccine post-listing period (01.01.2021–15.07.2021). It was found that during the first pandemic outbreak period (23.1.2020–08.04.2020), the public information needs for the COVID-19 vaccine were high, mainly focusing on the vaccine safety, vaccination necessity, adverse effects of vaccines, and the vaccine potency of coping with the variation of novel coronavirus. With the increasing number of COVID-19 patients in China following the first infection with COVID-19 in Wuhan, Hubei Province, China, the public experienced varying degrees of pandemic panic (Wang et al., 2021). To dispel their panic, the public became increasingly concerned with the COVID-19 vaccine in the hope that the vaccine could alleviate this severe novel coronavirus pandemic. They actively expressed information needs related to COVID-19 vaccines on online Q&A communities and portals so that the public information needs of COVID-19 vaccine were at a high level during the outbreak. Chinese and American experts indicated that the first vaccine would be available for clinical use by August 2020. However, since no vaccine has been developed for SARS, the Chinese public was worried about the safety and adverse effects of a COVID-19 vaccine developed within a short time. The novel coronavirus is an RNA virus with a high variation rate (Esakandari et al., 2020). Many highly infectious and high viral load strains have been developed, such as Alpha, Gamma, and Delta (Tracking SARS-CoV-2 Variants, 2021). The public asked many questions about the coping capacity of COVID-19 vaccines for new coronavirus variations, implying their information needs to be focused on the vaccine potency aspect. As the pandemic was gradually brought under control and the public gained confidence in the Chinese government's ability to prevent and control the pandemic outbreak, questions related to the necessity for vaccination began to increase among the majority of the public who did not intend to go abroad.

In the pre-listing vaccine period (09.04.2020–31.12.2020), the public information on the COVID-19 vaccine mainly concentrated on vaccine listing, R&D, and its safety. Although the COVID-19 pandemic in Wuhan has been successfully controlled during this period, several small-scale aggregated outbreaks emerged in other Chinese provinces and cities (Beijing, Hebei, Heilongjiang, Jilin). It has led to a great deal of renewed public interest in COVID-19 vaccine R&D and listing and needs questions about COVID-19 vaccine on online Q&A communities and portals.

During the vaccine post-listing period (01.01. 2021–15.07.2021), the public information needs of the COVID-19 vaccine focused on cautions before and after vaccination, evaluation of vaccine events, and adverse reactions. Due to individual differences, some people experience side effects of different degrees, such as local pain, rash, dizziness, etc. The public asked questions about this issue on online Q&A communities and portals to avoid side effects after vaccination. Furthermore, there were many social events related to the COVID-19 vaccine (vaccine patent exemptions and the vaccination rate of the public in other countries) during this period. The public also asked questions about the evaluation of events, especially for events closely related to themselves. The public seeks information about the COVID-19 vaccine by asking questions and is eager to receive feedback to further their understanding. Therefore, online Q&A communities and portals should provide dynamic and tailored information services according to changes in public vaccine information needs at different times to enhance service quality. For example, it is a good choice for online Q&A communities and portals to invite medical experts to answer questions and dispel doubts about meeting the current public information needs of the COVID-19 vaccine. Therefore, we constructed a general COVID-19 vaccine information need framework. It was found that the public information needed for vaccination was the most significant during the pandemic, accounting for half of all information needs. It included information on appointments, fees, vaccine type, population confirmation, preparation before vaccination, cautions after vaccination, and vaccination procedure. It reflects the public's desire to access relevant knowledge better to assist them in vaccination and minimize adverse reactions after vaccination. During the pandemic, the public information needed on the vaccine mechanism is the least. It indicated that the public was less concerned about the fundamental scientific principles of vaccines and how the vaccine works on the human body to acquire immunity.

By counting the information needs data of different groups, we found that the proportion of information needed regarding vaccination was high in all social groups. It was followed by relatively high information needs regarding vaccine R&D and listing among overseas students, airport workers, and healthcare workers, and relatively high information regarding vaccination necessity among teachers and domestic students. Overseas students, airport workers, and healthcare workers are susceptible groups that are easily exposed to COVID-19 and at a much higher risk of being infected than the general public. These groups are consequently more concerned about the COVID-19 vaccine R&D process and vaccine listing time, hoping to reduce their risk of infection and the chance of becoming seriously ill after vaccination (Lu et al., 2020). Therefore, the follow-up COVID-19 booster shots vaccination efforts should first be directed to these susceptible groups to ensure their safety at work. The relatively high need for information on vaccination necessity from teachers and domestic students reflects that with the successive local small COVID-19 pandemic outbreaks in China, the public is still at risk of contracting novel coronavirus. Therefore, government health departments should conduct vigorous promotion to improve the public's recognition of the importance of vaccination and willingness to vaccinate.

This study can effectively help the government and stakeholders improve information on service quality and make better health policies. At the beginning of the pandemic outbreak, the government and relevant organizations can grasp the public vaccine information needs in time through large-scale online forum data collection and analysis to get objective and accurate public opinion. It will enable them to timely formulate targeted health policies and solve the most concerned problems. For example, some fake news about mass adverse reactions to vaccination in China has sparked public concern, highlighting the need for public information about the COVID-19 vaccine's side effects. Chinese health departments found such information in time and combated misinformation by disclosing the scientific evidence for very low adverse reactions. In the middle stage of the pandemic, the government cyberspace administration, think tanks, information centers, libraries, and other stakeholders should build coordination mechanisms to further analyze and track online public data on social media for mining public vaccine information needs. To address such needs, timely and targeted health information should be released and pushed to the public through press conferences, traditional media, government official websites, social media platforms, and other channels (Liu and Ding, 2022). As the epidemic worsens, the abovementioned organizations should work together to conduct a dynamic analysis of public data to track the evolution of the public's vaccine information needs and adjust their health information service strategies and public health policies. For example, when the COVID-19 vaccine was licensed, considering the weak resistance of the elderly and infants, medical institutions did not provide vaccination for these groups. However, with the continuous improvement of vaccine safety and expansion of the scope of application, and more reports about the elderly and infant population infection, the vaccination information need of such groups is increasing. Government departments and medical institutions have been aware of such emerging information needs and promptly informed these people to get vaccination without delay, ensuring their health and safety.

## Conclusion

This study investigated the topic and distribution features of the public COVID-19 vaccine information needs. In addition, the topic features of public COVID-19 vaccine information needs in the different periods were examined. Data from Chinese online Q&A communities and portals were analyzed using K-means clustering and the LDA topic model. As a result, a general COVID-19 vaccine information need framework was constructed, including 8 main categories and 16 subcategories. It has several implications. First, online communities and portals should provide dynamic and tailored information services to realize organizational value according to changes in public vaccine information needs at different time points (Zhang et al., 2012; Day and Montoya, 2019). Second, the public information need regarding vaccination is prominent and should be addressed first. Third, government health departments should adopt an active pandemic prevention policy and prioritize susceptible groups, such as overseas students, airport workers, and healthcare workers, in the follow-up booster vaccination efforts. In a future study, data from microblogging platforms and other health social media should be collected and added to the dataset to analyze the public information needs of the COVID-19 vaccine comprehensively. In addition, other machine learning algorithms that have data clustering and topic extraction functions can be applied to better explore this issue.

## Data availability statement

Publicly available datasets were analyzed in this study. This data can be found here: https://www.zhihu.com/topic/19607469/hot.

## Author contributions

LW: conceptualization, methodology, writing-review and editing, and funding acquisition. ZX: formal analysis, data curation, visualization, and writing-original draft. TD: writing-original draft. All authors contributed to the article and approved the submitted version.

## Funding

Chinese National Social Science Key Funding “Chinese Information Poor People's Health Anxiety and Psychological Dredging under Healthy China Strategy” (Project Number: 21ATQ005) Chinese National Social Science Key Funding “Big Data-Driven Cloud Platform Construction and Intelligent Service of Science and Education Evaluation” (Project Number: 19ZDA348).

## Conflict of interest

The authors declare that the research was conducted in the absence of any commercial or financial relationships that could be construed as a potential conflict of interest.

## Publisher's note

All claims expressed in this article are solely those of the authors and do not necessarily represent those of their affiliated organizations, or those of the publisher, the editors and the reviewers. Any product that may be evaluated in this article, or claim that may be made by its manufacturer, is not guaranteed or endorsed by the publisher.
